# Friend and foe: factors influencing the movement of the bacterium *Helicobacter pylori* along the parasitism–mutualism continuum

**DOI:** 10.1111/eva.12231

**Published:** 2014-11-20

**Authors:** Derek Lin, Britt Koskella

**Affiliations:** 1 Berkeley, CA, USA; 2 Biosciences, University of ExeterTremough, UK

**Keywords:** antibiotic resistance, carcinogenic bacteria, gut microbiota, host–parasite coevolution, human health, microbiome, probiotics, virulence factors

## Abstract

Understanding the transition of bacterial species from commensal to pathogen, or vice versa, is a key application of evolutionary theory to preventative medicine. This requires working knowledge of the molecular interaction between hosts and bacteria, ecological interactions among microbes, spatial variation in bacterial prevalence or host life history, and evolution in response to these factors. However, there are very few systems for which such broad datasets are available. One exception is the gram-negative bacterium, *Helicobacter pylori,* which infects upwards of 50% of the global human population. This bacterium is associated with a wide breadth of human gastrointestinal disease, including numerous cancers, inflammatory disorders, and pathogenic infections, but is also known to confer fitness benefits to its host both indirectly, through interactions with other pathogens, and directly. Outstanding questions are therefore why, when, and how this bacterium transitions along the parasitism–mutualism continuum. We examine known virulence factors, genetic predispositions of the host, and environmental contributors that impact progression of clinical disease and help define geographical trends in disease incidence. We also highlight the complexity of the interaction and discuss future therapeutic strategies for disease management and public health in light of the longstanding evolutionary history between the bacterium and its human host.

## Introduction

The human-specific bacterium, *Helicobacter pylori,* infects an estimated 50% of the world's adult population, with the highest prevalence in industrially underdeveloped areas (Kauser et al. 2004). Genetic evidence suggests *H. pylori* as one of the oldest and most common occupants of the human gut microbiome, arriving before the migration out of Eastern Africa over 60 000 years ago (Linz et al. 2007; Ahmed et al. 2009; Plottel and Blaser 2011). Despite this long association, substantial histologic, epidemiologic, and experimental data suggest *H. pylori* is a causative agent of life-threatening diseases such as gastric adenocarcinoma (GAC) and gastric MALT Lymphoma, as well as their precursory gastric lesions such as chronic gastritis, gastric atrophy, intestinal metaplasia, and dysplasia (Crew and Neugut 2006). A long-term observational study (mean follow-up: 7.8 years) of 1246 *H. pylori -*positive and 280 *H. pylori -*negative patients, including those with active duodenal ulcers, active gastric ulcers, gastric hyperplastic polyps, or nonulcer dyspepsia, found 36 cases (2.9%) of gastric cancer in the *H. pylori* -positive cohort with no cases of gastric cancer in the *H. pylori* -negative cohort (Uemura et al. 2001). Despite well-documented carcinogenic properties, *H. pylori* infection is pervasive both through time and across space. It is therefore possible that the bacterium confers a benefit to its host under some circumstances and is therefore maintained in the human population. Some evidence for such a benefit exists (Table1); it has been suggested that *H. pylori* confers protection against tuberculosis (TB) through induction of antagonistic interferons for the causative agent, *Mycobacterium tuberculosis,* and that it plays a role in reducing the risk of esophageal adenocarcinoma (EAC), gastroesophageal reflux disease, stroke, lung cancer, asthma, allergies, and irritable bowel disease (Blaser 1999; Ahmed et al. 2009; Luther et al. 2010; Perry et al. 2010; Plottel and Blaser 2011; Walker and Talley 2014). Antibacterial activity against other virulent bacterial species has also been documented, showing a preventative effect against diarrheal diseases and an augmented enteral and systemic response to *Vibrio cholerae*, the causative agent of the disease cholera (Blaser and Berg 2001; Cohen et al. 2012; but see Clemens et al. 1995; Shahinian et al. 2000).

**Table 1:** Examples of current evidence in support of both harmful and beneficial effects correlated with *Helicobacter pylori* infection.

Effect of infection	Affect on health	Citation
Increased prevalence of gastric cancer	(−)	Uemura et al. (2001), Kodaman et al. (2014)
Increased risk of infection	(−)	Shahinian et al. (2000),
with *Vibrio cholera,* and risk of progressing to severe cholera	(−)	Clemens et al. (1995)
Increased prevalence of peptic ulcers	(−)	Maeda et al. (1998)
Chronic and acute gastritis	(−)	Graham et al. (2004)
Suppression of bacteria causing tuberculosis	(+)	Perry et al. (2010)
Reduced risk of eczema	(+)	Amberbir et al. (2011)
Reduced risk of gastroesophogeal reflux disease	(+)	Sonnenberg et al. (2010)*
Protection against diarrheal diseases	(+)	Cohen et al. (2012)
Reduced risk of esophageal cancer	(+)	Islami and Kamangar (2008)
Reduction of asthma and allergy	(+)	Chen and Blaser (2007)
Reduced risk of irritable bowel disease	(+)	Luther et al. (2010)

* But see Qian et al. (2011).

**Figure 1:**
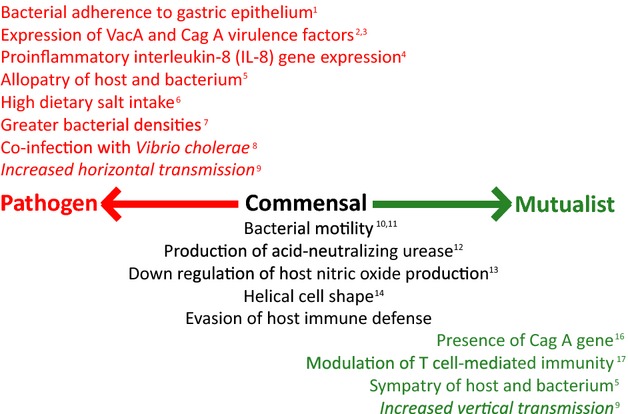
Schematic summarizing the key genetic, ecological, and evolutionary factors known to influence the transition of *Helicobacter pylori* along the parasitism–mutualism continuum. Factors known to increase potential pathogenicity are in red, those that are involved in colonization and survival in the human host, but which have no direct evidence of conferring either cost or benefit to the host are in black, and factors that are thought to be associated with conferred benefits to the host (either directly or indirectly) are in green. Further factors that are predicted from theory but which have not been examined empirically are included in italics. Details of each association are discussed within the main text. References: (1) Oleastro and Ménard (2013); (2) Censini et al. (1996); (3) Maeda et al. (1998); (4) Persson et al. (2011); (5) Kodaman et al. (2014); (6) Gaddy et al. (2013); (7) Atherton et al. (1996); (8) León-Barúa et al. (2006); (9) Anderson and May (1982); (10) Eaton et al. (1992); (11) Ottemann and Lowenthal (2002); (12) Tsuda et al. (1994); (13) Gobert et al. (2001); (14) Bonis et al. (2010); (15) Salama et al. (2013); (16) Chen and Blaser (2007); (17) Arnold et al. (2011).

A better understanding of the factors that move this potential pathogen along the parasitism–mutualism continuum is key for effective detection, management, and disease prevention (Fig.1). This is especially true given the emerging interest in microbiome health and the developing datasets aiming to uncover the composition of ‘healthy’ human microbiota across the population (Bäckhed et al. 2012). Our understanding of this bacterium continues to be confounded by uncertainties surrounding mode of transmission, environmental influences, effects of host genotype, and complex ecological interactions within the microbiome (e.g. Rolig et al. 2013). In this review, we aim to explore the interactions between *H. pylori* bacteria and their human hosts by considering trends in geographic variation, association with disease, genes underlying virulence, interactions with other microbes, and the influence of host immune response. We then highlight future directions for management of *H. pylori* -associated disease as it relates to preventative public health.

## Factors known to contribute to disease development

As one of few organisms to be classified as a ‘group 1 (definite) carcinogen’ by the International Agency for Research on Cancer, colonization by *H. pylori* has a major etiological association with increased risk of several gastric disorders including gastric cancer, which accounts for 10% of all cancers worldwide and over 700 000 deaths every year (Ahmed et al. 2009; Kodaman et al. 2014). Only a small subset of infections (<1%) develops into gastric cancer due to, among other things, the range of alternate factors contributing to severity of infection (Fig.1; Kodaman et al. 2014), some of which include bacterial polymorphism, genetic predisposition, diet, and socioeconomic-related living conditions. In this section, we will explore what is currently known about the many interacting factors underlying *H. pylori* -associated disease.

### Virulence factors

The majority of *H. pylori* infections are benign, and the development of clinical disease depends on a variety of factors such as adherence of the bacterial cells to the gastric epithelium (Oleastro and Ménard 2013) as well as some not yet well-understood genetic, epigenetic, and environmental factors (Wroblewski et al. 2010), but primarily depends on the presence of two different genes, the cytotoxin-associated gene A (*CagA*) and the vacuolating cytotoxin (*VacA*) (Ahmed et al. 2009). Motility of *H. pylori* cells in the stomach lining is common, but several different outer membrane proteins facilitate the binding of bacterial cells to gastric epithelial surfaces (Algood and Cover 2006). This binding has been linked to evasion from the host immune system and long-term persistence, as well as increased delivery of the *CagA* oncoprotein into gastric cells, all of which are linked to disease (Oleastro and Ménard 2013). Virulence factors such as flagella and stomach acid-neutralizing urease are also common attributes of all *H. pylori* strains. Knockout mutants of the flagellar or urease genes are unable to colonize gnotobiotic piglets or other animal models (Eaton et al. 1992; Tsuda et al. 1994; Covacci et al. 1999; Ottemann and Lowenthal 2002; Algood and Cover 2006).

The bacterial polymorphisms considered to be the most important contributors to *H. pylori* cytotoxicity and carcinogenicity are those found within the *CagA* gene of the *cag* pathogenicity Island (CagPAI), a set of genes coding for a type IV secretion system, and those influencing the activity of *VacA* genes, a secreted protein toxin responsible for gastric epithelial erosion (Censini et al. 1996; Ahmed et al. 2009; Duncan et al. 2013). For example, activity of the *VacA* gene was found in 16 of 24 (67%) patients with peptic ulcers but in only 16 of 53 (30%) patients without (Maeda et al. 1998). Although *VacA* is present across *H. pylori* strains, it is estimated that only 50% of all western strains secrete vacuolating cytotoxin (Figura et al. 1989), one of the factors that leads to the observed lower rate of clinical infection in western countries. This marker is therefore of little use for predicting pathogenicity of circulating strains, as it seems to be the expression of the gene, rather than any particular mutation, that is important in shaping the outcome of disease. This also suggests the ease at which this bacterium might be able to respond to the host environment and alter its phenotype accordingly.

*CagA* is the gene most strongly associated with highly carcinogenic strains (Bourzac and Guillemin 2005), and the interplay between the *CagA* and *VacA* loci has proven to be an important contributor to the development of gastritis and GAC (Matos et al. 2013; Rahimian et al. 2014; but see Xiang et al. 1995). *CagA* is thought to only be present in approximately 60% of *H. pylori* strains and, unlike *VacA*, is almost always expressed when present (Maeda et al. 1998). Serum anti-*CagA* antibodies were found in 165 of 197 (84%) patients with duodenal cancer, but in only 25 of 45 (56%) patients with functional dyspepsia. This marker is therefore a useful candidate for monitoring and predicting disease, although clearly the association is not tight enough to be used in diagnosis. In this case, therefore, bacterial genetics plays a clear but not singular role in pathogenicity. Opposed to the increased risk of gastric cancer, recent evidence and a meta-analysis of 19 different studies suggest *CagA*-positive strains are also associated with a decreased risk of EAC, asthma, and allergies (Islami and Kamangar 2008; Chen and Blaser 2007; Pacifico et al. 2014). The potential protective effect of *H. pylori* against asthma has also been experimentally confirmed in a mouse model (Arnold et al. 2011). Again, this work highlights the extreme difficulty associated with predicting the outcome of disease in patients harboring *H. pylori* infection, given the great variation and somewhat weak associations observed.

*H. pylori's* causative and protective roles with GAC and EAC, respectively, are further evidenced by a marked decline in the prevalence of both *H. pylori* and GAC in the developed world, but a rise in EAC such that it now makes up approximately half of all esophageal cancers in countries such as the United States and the United Kingdom (Islami and Kamangar 2008). This rise in EAC strongly correlates to the disappearance of both *H. pylori* and peptic ulcers from the developed world over the last several decades; although risk factors such as alcohol consumption, tobacco, and obesity need also be considered (Islami and Kamangar 2008; Whiteman et al. 2008), as does the widespread dissemination of antibiotics, whether for agricultural or medicinal purposes, that contributes to the shifting composition of the gut microbial community (Jernberg et al. 2010; Zeissig and Blumberg 2014). It is theorized that urease activity in *H. pylori* neutralizes the stomach's acidity and consequently protects against EAC by lowering the risk of acid-reflux disease; but due to the complex nature of the microbiome, further research is necessary to define the role of *H. pylori* and differentiate it from simply a biomarker for the decline of other beneficial microbial species.

Given the potential for harm, and the long evolutionary association between *H. pylori* and its human host population, we should expect mechanisms of both human immune responses to the bacterium and of counter adaptation by the bacterium to evade the host immune response. Indeed, *H. pylori* is known to have several strategies to avoid antigenic recognition through phase and surface protein variation, and certain strains are also capable of emulating host cells through the use of the surface proteins pbgA and pbgB that bind plasminogen, thereby coating themselves with this host protein and making them undetectable as foreign agents (Jönsson et al. 2004; Algood and Cover 2006). Other strategies for avoiding detection involve the O-antigen of the *H. pylori* lipopolysaccharide (LPS), a potent signaler of inflammatory response; the majority of *H. pylori* tested worldwide have LPS that mimic Lewis blood group antigens (Simoons-Smit et al. 1996; Algood and Cover 2006). The LPS is a common pathogen-associated molecular pattern (PAMP) and a major component of the outer membrane of bacterial cells that can be recognized by the innate immune system of hosts. Portions of the LPS are highly conserved across *H. pylori* strains, but there has also been good evidence for high levels of phase variation of this antigen within patients (Appelmelk et al. 1998). Specifically in the Lipid A region, the portion of the LPS recognized by host immune receptors, there is a large amount of within-host variation presumably as a result of its versatility in adapting to the specific host conditions (Suarez and Peek 2014). The potential role of such within-host variation in causing disease has yet to be tested.

The ability of *H. pylori* to evade detection and subsequent elimination by the host immune response (Salama et al. 2013) likely contributes to its sustained occupation of the human gut. The resistance of many *H. pylori* strains to immunological elimination may be maintained by its high recombination rate (rate at which recombination events start at any particular nucleotide, *r *=* *6.9 × 10^−5^) and mutation rate (μ* *= 4.1 × 10^−5^), for example relative to *E. coli* (*r *=* *7 × 10^−12^; μ* *= 3 × 10^−10^) (Falush et al. 2001; Kennemann et al. 2011). However, recent work examining isolates collected from the same patients over time, as well as individuals from the same families, suggests that substitutions are three times more likely to have been introduced by recombination than mutation (Morelli et al. 2010). This work also demonstrates that while the introduction of mutations is frequent, the rate of substitution over longer time periods is many fold lower, in part due to purifying selection acting on nonsynonymous mutations. Furthermore, while *H. pylori* is known to constitutively express the mismatch repair pathway, it has a distinct response to DNA damage. The most common bacterial response to DNA damage is the SOS response, which typically includes transcriptional induction of DNA repair systems and a temporary halt to cell division. However, *H. pylori* lacks this pathway and responds to stress by inducing natural competence (i.e., the uptake of DNA from the environment; Dorer et al. 2010). This elevated recombination rate increases *H. pylori* variability and potential for adaptation to the host environment (Suerbaum and Josenhans 2007). Isolates from unrelated host individuals are different enough for *H. pylori* to be considered a ‘quasi-species’, the majority of mutations occurring in the third base of codons, therefore giving a diverse genetic fingerprint while conserving 93% of genomic protein sequences between strains (Covacci et al. 1999). Independent isolates often vary from one another by 3–5% of base substitutions, even in essential housekeeping genes, which is a level of variation not often seen in other bacterial pathogens (Datta et al. 2003). Much of this genetic diversity could be expected from the long evolutionary history of the association and the high mutation rate, but given the rate of divergence observed even across hosts from similar geographical locations, it is more likely that there exist many circulating strains with slightly different phenotypes and/or that this variation has great adaptive potential for the bacterial population. In particular, the known virulence-associated genes, *VacA* and *CagA*, are more likely to contain nonsynonymous variation, suggesting selection pressure on these proteins to vary functionally and structurally to avoid recognition (Datta et al. 2003). Together, this high unexplained variation and somewhat weak association between bacterial genome and disease suggest that although genetics is likely to be a strong contributing factor to disease emergence and progression, alternate factors such as the host genotype, environment, or interactions within the microbiome are also likely to play a key role.

### Genetic predisposition of hosts

Addressing the host genetic component of clinical infection must be performed in the context of geographical location, socioeconomic environment, and cultural practices. A prime example of epidemiological complexity in relation to *H. pylori* is found in a study of three ancestrally, and geographically, distinct communities in West Bengal: the Oroan, the Santhal, and the ethnic Bengalis (Datta et al. 2003). The lineages of the Oroan (ethnically proto-Australoid) and the Santhal (ethnically Dravidian) are ethnic minorities representing ≈5% of the population with only rare intermarriage with other ethnic groups, as evidenced by distinct autosomal and mitochondrial DNA markers. These communities are similar in socioeconomic status, with relatively low levels of hygiene and sanitation, making them good communities for studying *H. pylori* infection, which is abundant. Despite cultural, geographical, and genetic isolation, PCR analysis of *CagA* and *VacA* in *H. pylori* from across the groups revealed that strains from different ethnic groups share similar frequencies of chosen genetic markers. This similarity, in conjunction with widespread metronidazole resistance (an antibiotic used in mainstream communities since the 1970s but not used by the Oroan or Santhal), argues for close relatedness between strains of *H. pylori* and recent transmission into and between groups. Similar abundance of *H. pylori* infection and of *CagA+*/*VacA*+ strains would be expected to correspond to similar abundance of *H. pylori* -associated gastrointestinal disease between groups. However, this was not found to be the case. Although clinical infection is common among ethnic Bengalis (representing the ethnic majority and believed to have Indo-European ancestry), it is virtually nonexistent in the cohorts from the two ethnic minorities. This distinction suggests several different, but not mutually exclusive, possibilities: [I] as host inflammatory response to *H. pylori* varies with host genotype (Datta et al. 2003), these genetically distinct host populations could be differentially reactive to the same enteric pathogens; [II] subtle mutational differences, such as point mutations undetected by the study, among strains might play a significant role in determining expression and potency of virulence genes; or [III] due to the predicted positive correlation between transmission frequency and virulence (Bremermann and Pickering 1983), it is possible that larger communities with higher population density facilitate higher transmission frequency and, in turn, select for increased virulence (Anderson and May 1982), although one argument against this theory includes high incidence of gastric cancer in less dense rural populations (Ma et al. 1998).

In the case of *H. pylori*, geographical differences in infection might be better explained by socioeconomic, genetic, and cultural factors than by physical location, but multiple uncontrolled variables make it impossible to attribute onset of clinical infection to a single factor. Although the specific case from communities in West Bengal cannot definitively implicate ethnicity as a component of *H. pylori* infection, another study found a fourfold higher bacterial colonization rate among African-Americans compared to Caucasians within the same community and socioeconomic class (Malaty et al. 1999). This 12-year follow-up study of 212 children found 40% of the African-American cohort was infected with *H. pylori* by age 7, compared to 11% of the Caucasian cohort, with 50% of the Caucasian cohort eliminating infections over the 12-year period, and only 4% of African-Americans eliminating the bacteria from their microbiota. Although the children in the study attended the same schools, the researchers did not take into account social behavior, customs, or dietary habits.

Current research on host polymorphisms in inflammatory response genes supports the role of host genetics in determining predisposition to gastric cancer (GC). *Helicobacter pylori* -induced GC is a product of a chronic inflammatory response, and differing host responses likely determine the outcome of *H. pylori* infection. One meta-analysis of 62 studies, conducted on a total of 24 577 patients with GC worldwide, including 9905 *H. pylori* -positive patients and 14 672 *H. pylori* -negative patients, uncovered a clear role for host genetics in the development of GC (Persson et al. 2011). By studying single nucleotide polymorphisms (SNPs) in the host interleukin (IL) genes, inflammatory cytokines mediating gastric physiology and pathophysiology, investigators were able to identify several high-risk polymorphisms, one of the most significant being a polymorphism in the proinflammatory interleukin-8 (IL-8) gene (Persson et al. 2011). IL-8 transcription leads to increased oxidative stress on the gastric mucosa, is produced as a response to *H. pylori*, and is upregulated by *CagA* expression (Yamaoka et al. 1999a,b). Furthermore, a genomewide association study (GWAS) in Japan identified the prostate stem cell antigen (PSCA) gene, a glycosylphosphatidylinositol-anchored cell surface antigen, as a gastric cancer-susceptibility gene, and the Mucin 1 (MUC1) gene, a cell membrane bound mucin protein, as risk factors for gastric cancer (Saeki et al. 2013). However, this latter study did not explicitly test for an interaction between *H. pylori* infection and the genes identified as risk factors.

To support the idea of host genome-by-bacterial genome interaction in determining clinical infection, a recent study run in Colombia concluded that differences in severity of gastric lesions could be explained by coevolution between human and *H. pylori* populations (Kodaman et al. 2014). Host genetics in this region are shaped by migration and mating among populations of European, African, and Amerindian ancestry. Admixture analysis of high-density human genotypic data revealed a predominantly African ancestral cluster in the low-risk, coastal populations, with similar proportions of Amerindian and European ancestry; whereas the high-risk, mountain populations were predominantly Amerindian, with a significant proportion of European ancestry and negligible African ancestry. Congruously, all strains of *H. pylori* in this study also contained the genetic signatures of European, African, and Amerindian backgrounds. However, coastal isolates were predominantly African in ancestry, whereas mountain isolates were predominantly European. The authors suggest the relatively benign infections observed in coastal populations can be attributed to the matching of predominantly African ancestry of both host and bacteria. The more severe infections of mountain populations are suggested to be a product of mismatch between ancestrally Amerindian hosts with European strains. In this case, the interaction effect was found to be five times higher than the main effect of *CagA* identity, leading the authors to theorize that higher bacterial fitness (in terms of growth rate) in allopatric strains could lead to a competitive advantage over the indigenous strains. These findings are in accordance with evolutionary theory, which predicts that chronic pathogens with vertical transmission will become less virulent over time through coevolution, and could help explain the ‘African Enigma,’ or the exceptionally low incidence of gastric cancer in Africa despite very high rates of *H. pylori* infection (Holcombe 1992). Clinical infection and the shift to parasitism by *H. pylori* might therefore primarily be a product of mismatched host and parasite genomes; however, further research needs to be performed to determine whether this is a localized phenomenon or applicable to all populations, especially in light of gene flow among populations.

### Dietary practices

Regional and cultural differences in dietary practices likely contribute to both the rate of *H. pylori* infection and the severity of infection. For example, one study performed in Pune, India, identified lower socioeconomic status, meat consumption, smoking, eating restaurant food, and drinking nonfiltered or nonboiled water as risk factors for infection (Mhaskar et al. 2013). Additionally, meat/fish consumption and family history of peptic ulcer disease were found to be risk factors for peptic ulcer disease, while parasite infection and chili pepper consumption were indicative of protective effects. High-salt diets have recently been found to exacerbate *H. pylori* infection by upregulating transcription of *CagA* and increasing the risk of GAC; the same results were found for low-iron diets (Gaddy et al. 2013). However, as both meat/fish and high-salt diets have also been associated with a wealth of other health risks that decrease host fitness, it is unclear whether their effect on *H. pylori* growth and virulence is direct or indirect.

One 15-year follow-up study of *H. pylori-*infected individuals found evidence that both garlic and vitamin intake (of E, C, and selenium) were ineffective at preventing incidence of gastric cancer, although there was some association with decreased mortality (Ma et al. 2012), and there is some evidence that diets high in fruits and vegetables can decrease risk of *H. pylori* infection (Brown 1999). For example, one recent study found diets of broccoli sprouts rich in sulforaphane (a compound with powerful bactericidal properties) were able reduce *H. pylori* colonization and gastric inflammation relative to sulforaphane-poor alfalfa sprouts; these effects are predicted to be both direct via antibacterials and indirect through increased mammalian cytoprotective response (Yanaka et al. 2009). Additional research has explored dietary supplementation and demonstrated anti- *H. pylori* properties observed *in vivo* from green tea, liquorice, and honey; red wine provided minor symptomatic relief and use of propolis resulted in conflicting results (Ayala et al. 2014). More research is now needed not only on the combined effects of these supplements in long-term maintenance of *H. pylori* infection but also in determining the mechanistic link between diet and infection, as well as diet and progression of *H. pylori -*associated disease. Elimination of nickel from the diet also increases the success rate of *H. pylori* eradication when used in conjunction with triple antibiotic therapy; eradication increased from 12 of 26 individuals (standard diet) to 22 of 26 individuals (nickel free) (Campanale et al. 2014). The authors attribute this to disruption of urease and NiFe-hydrogenase, two critical nickel-containing enzymes in *H. pylori*. In terms of additional environmental/behavioral factors known to influence susceptibility, there is evidence that use of well water, use of pit latrine, less frequent boiling of drinking water, and infrequent hand wash practice after toilet use and before meals act as high-risk factors for *H. pylori* infection, while several regionally specific foods have also been found to be protective (Lee et al. 2012). These environmental/behavioral risk factors are not surprising, as *H. pylori* is known to be transmitted through oral–oral and fecal–oral routes.

### Length of infection

Among the factors known to influence the transition from *H. pylori* infection to progression of clinical disease is the acquisition and elimination of the bacterium in early childhood and the length of infection (Pounder and Ng 1995; Ma et al. 1998; Brown 1999). Incidence of gastric cancer has been shown to be higher in areas where childhood infection is common, while duodenal ulcers are a more common outcome of adult onset infections (Brown 1999). Trends in *H. pylori* infection are also associated with a country's relative stage of development. According to the Human Development Index (HDI), the majority of children from ‘underdeveloped’ or ‘developing’ countries become infected during childhood and maintain chronic infection throughout life (Brown 1999; Kusters et al. 2006). One study from the Shandong Province in China found that children ages 5–6 and adults have comparable rates of infection at approximately 70% (Ma et al. 1998), implying infection is maintained throughout life. In contrast, only a minority of children from ‘developed’ countries becomes infected, and infection frequency increases proportionally with age (Pounder and Ng 1995; Kusters et al. 2006). A Canadian review of *H. pylori* prevalence found that first generation individuals who immigrated as adults (>20 years of age) had a rate of infection, and a rate of gastric cancer consistent with their country of origin, with an odds ratio of 9.7 times that of second generation individuals. These results paralleled US studies (Jones et al. 2012). As spontaneous elimination is uncommon, it is not surprising that infection in immigrants reflects that of their native country, but together, these results argue that transmission dynamics are specific to the region in question.

Spontaneous elimination of *H. pylori* by the immune system is more common in children but rarely observed in adults (Kusters et al. 2006), only occurring in 0.1–1.1% of infections annually (Pounder and Ng 1995; Brown 1999). Therefore, early childhood infection typically results in an extended length of infection and likely affords more opportunities for oncogenic mutations, although a defined role for *H. pylori* in tumorigenesis is still actively debated, with several studies supporting a range of theories. Two possible pathways are suggested for gastric tumorigenesis: one assigns carcinogenic activity to *H. pylori* itself, while the other describes the establishment of a carcinogenic environment due to long-term infection (Mobley et al. 2001). Both mechanisms involve the initial complication of superficial gastritis progressing into atrophic gastritis, which eventually develops into intestinal metaplasia, a precursor for gastric cancer. Other theories outline the exact mechanism of tumor formation. For example, one model invokes the recruitment of stem cells from the bone marrow observed during *H. pylori* -mediated gastric inflammation (Houghton et al. 2004), while another proposes that chronic *H. pylori* -induced gastric inflammation makes the niche inhabitable, eliminates *H. pylori*, and supports other cancer-promoting microbial species (Plottel and Blaser 2011). This latter theory is contradicted by prevention of gastric carcinoma from prophylactic eradication of *H. pylori* after detection of early gastric cancer (Fukase et al. 2008), but this discrepancy may be indicative of the different modes of tumorigenesis that depend on cancer type. Interestingly, there is currently a lack of evidence showing any connection between immunodeficiency, such as acquired immunodeficiency syndrome, and uncontrollable *H. pylori* infections, despite this being a common situation with other pathogens (Pounder and Ng 1995). This is unsurprising in context of *H. pylori*'s ability to evade host immune defenses and supports the theory that chronic inflammatory response over a lifetime is mainly responsible for promoting gastric cancer.

### Transmission dynamics

As a bacterium detectable throughout the world, *H. pylori* is capable of colonizing a wide spectrum of host gastrointestinal niches and microniches (Blaser and Atherton 2004). Studies on habits that contribute to transmission have been inconsistent in their results and vary depending on investigators and cohorts. Overall, proper nutrition is associated with a decrease in transmission, while factors associated with a lower socioeconomic status (i.e. inadequate sanitation, high-density living spaces, and contaminated food and water) and eating raw or uncooked vegetables facilitate transmission (Brown 1999). Although the exact mechanism of transmission is unknown, infection is highly correlated to human crowding and poor sanitation (Jones et al. 2012). Discrepancies in transmission routes are attributed to the difficulty in culturing oral, gastric, and fecal isolates and the resulting lack of data, but the predominant theory involves direct human-to-human transmission with evidence for both oral–oral and fecal–oral transmission (Brown 1999). One study of 289 adults and 98 children in a Chinese village with an exceptionally high risk of stomach cancer revealed an infection rate of 89% for children with at least one infected parent and only 22% for children with no infected parents (Ma et al. 1998), suggesting a primarily vertical mode of transmission. Some *H. pylori* infections are conserved to the point where children have similar strains as their parents and will maintain those strains after starting their own family; oddly enough, however, husbands and wives have been shown to not typically exchange strains (Covacci et al. 1999). Familial/vertical transmission of parasites is predicted to select for reduced virulence over time (Anderson and May 1982), which may be reflected in the relative tolerance to *H. pylori* by most human hosts. However, the occurrence of horizontal transmission (especially given the increased movement of recent humans) may well negate any such selective pressures.

Socioeconomic factors play a distinct role in transmission due to differences in waste management, hygiene, and the likelihood of practices such as premastication by parents and sharing of eating utensils such as chopsticks (Dowsett and Kowolik 2003). *Helicobacter pylori* has been detected in oral cavities, fecal samples, houseflies, and under fingernails, and it is suggested that each of these are possible routes of transmission, although the likelihood of each differs regionally (Dowsett and Kowolik 2003; Kusters et al. 2006). The body of research on whether oral *H. pylori* is viable as a gastric pathogen offers conflicting evidence and obscures the oral–oral route of transmission. However, there is a possibility that nonviable oral *H. pylori* can still participate in horizontal gene transfer with existing gastric colonies, diversifying the infection, especially when the high recombination frequency among strains is taken into account (Kennemann et al. 2011). Horizontal transmission is likely to increase in unsanitary, highly crowded environments, making multiple infection and recombination among strains more common in dense populations (Jones et al. 2012; Kodaman et al. 2014). Increased horizontal transmission may help explain why certain regions exhibit high rates of gastric cancer despite evidence of predominantly vertical transmission for the system as a whole.

Although the association between *CagA* status and bacterial density *in vivo* has had conflicting support, *CagA*-positive strains are known to trigger high levels of interleukin production, whereas *CagA*-negative strains trigger little to no interleukin production (Atherton et al. 1996; Yamaoka et al. 1999a,b). Bacterial density has been shown to be directly related to severity of disease, with higher density in duodenal ulcers than in gastritis (Atherton et al. 1996), and it has been suggested that the increased inflammation associated with *CagA*-positive strains may also be associated with higher transmission rates between hosts (Blaser 1997). The fact that *CagA* frequency varies geographically, with more *CagA*-positive strains in East Asia (making up approximately 90% of all isolates [Saribasak et al. 2004; Duncan et al. 2013]) than in western countries, suggests the possibility of a fitness trade-off preventing *CagA*-positive status from spreading to fixation globally. One possibility is that the increased transmissibility is a function of increased growth rate within the host and that this growth rate explains both increased spread and increased harm to the host. Classic evolutionary theory on the virulence-transmission trade-off suggests that while increased within-host growth rate should increase bacterial fitness through transmission success, there is also a cost in terms of host death rate (Anderson and May 1982; Bremermann and Pickering 1983; May and Anderson 1983; Ewald 1987; Frank 1996). As such, while *CagA*-positive strains may benefit from easier communicability, their increased virulence is likely to decrease the time frame during which they are communicable by the host; *CagA*-negative strains may benefit from a longer host life and therefore a longer period of communicability. However, the direct application of such classic evolutionary theory should be made with caution given the difference in generation time between the host and the bacterium. For example, it is unclear whether the shortened life span of hosts is sufficient to impose meaningful selection pressure on the pathogen population. Indeed, many of the diseases associated with *H. pylori* infection occur later in life. This potentially relaxes selection on the bacterial population, but also alters the response to selection of hosts; the more likely onset of *H. pylori* -associated cancers in postreproductive hosts could mean little to no response to selection in the human population (Greaves 2007). Further theoretical work and system-specific modeling could help inform predictions regarding the response of both bacteria and host population to selection mediated by the other.

Transmission of *H. pylori* is also influenced by interactions within the other bacteria, both pathogens and commensals. Contaminated water and poor community hygiene, both indicators of socioeconomic status, are known risk factors for *H. pylori*, as well as *V. cholerae*. Interactions between these gastrointestinal pathogens increase the severity of the disease and consequently alter transmission dynamics. In one study of 75 *H. pylori* -positive patients from Peru, 35 of which had cholera-induced dehydration and 40 of which had no history of cholera, the patients with cholera were found to have significantly more chronic atrophic gastritis (45.7% vs 12.5%; *P *= 0.002) and intestinal metaplasia (37.1% vs 2.5%; *P *< 0.01) in the gastric body than those without cholera (León-Barúa et al. 2006). Both chronic atrophic gastritis and intestinal metaplasia result in inflammation of the stomach and increase the risk of gastric adenocarcinoma (Stemmermann 1994; Giannakis et al. 2008). The observed inflammation aligns with the findings that orally administered *V. cholerae* vaccines signal the recruitment of antibody secreting cells to the gastric mucosa only in patients with active *H. pylori* infections (Mattsson et al. 1998). In individuals lacking vibriocidal immunity, *H. pylori* infection was associated with a significant increase in risk of life-threatening cholera (Clemens et al. 1995). *Vibrio cholerae-*induced diarrhea eases *H. pylori* transmission through the fecal route, which minimizes the cost of increased host inflammatory response. Data such as these highlight one of the many unknown interactions *H. pylori* potentially has with other bacterial species, pathogenic or not, that contribute to both transmission and virulence.

### Geographic variation of infection

The long coevolutionary history between humans and *H. pylori* is intimate enough for *H. pylori* genotyping to distinguish between closely related human groups. As a symbiotic accompaniment of human migration out of Africa, DNA analysis of *H. pylori* can accurately reflect population movement and gene flow between locally distinct populations; this is in part due to its primarily vertical transmission, which creates a global genetic structure paralleling that of humans (Wirth et al. 2004). The use of transcriptomics to examine global gene expression across isolates has further underpinned evidence of phylogeographic variation among strains (Sheh et al. 2013). By coculturing clinical isolates with gastric epithelial cells, transcriptional profiles could be compared across strains from regions with low and high risks of GC, uncovering clear transcriptional differences among genes associated with motility and pathogenicity, including upregulation of *CagA* and *VacA* in ancestrally European strains. The full explanation of why *CagA*-positive/negative strains vary geographically is still unknown. Korea and Japan have the highest rates of GC worldwide, which is in part due to a high incidence of *H. pylori* infections relative to other developed countries in North America and Europe (Saeki et al. 2013). Interestingly, Japan has a high rate of GC (age-standardized incidence rate of 62.7/100 000), but a lower rate of *H. pylori* seroprevalence (39.3%) than other countries such as Bangladesh (92%) and India (79%), which exhibit much lower rates of GC with 1.6/100 000 and 5.7/100 000, respectively. These geographic differences are likely explained by the interaction between *H. pylori* and host genetics, as well as environmental influences. In agreement with this expectation, one study uncovered two high-risk host genotypes and one potentially protective genotype within the Japanese population; 95% of the population was found to have one of the two risk genotypes and 56% were found to have both, while there was an exceptionally low frequency of the protective genotype (Saeki et al. 2013). These risk phenotypes coincide with findings that most Japanese strains of *H. pylori* produce both *CagA* and *VacA* (Maeda et al. 1998). There is a clear role of susceptible host genotypes in shaping the observed trends in geographic variation of infection outcome, but the ability to generalize such a pattern beyond this population is yet to be determined.

Geographic trends in disease incidence are well matched by geographic trends in *H. pylori* virulence genes. For example, a study of 335 *H. pylori* DNA samples from 8 countries worldwide (Spain, Peru, South Africa, England, Ireland, Japan, Costa Rica, and India) found considerable variation in the organization of the *cag*-PAI (Kauser et al. 2004). PCR analysis revealed a high prevalence of gene rearrangements within the *Cag*PAI, more so than any other region on the chromosome. The *Cag*PAI was disrupted in the majority of patient isolates throughout the world, with 57.1% of Japanese isolates exhibiting the highest occurrence of intact *Cag*PAI followed by Peruvian (18.6%) and Indian (12%) isolates (Kauser et al. 2004). One Turkish study of 65 *H. pylori* -positive patients (22 with gastritis, 33 with peptic ulcer disease, and 10 with gastric cancer) discovered a higher frequency of *CagA*-negative isolates that more closely resembled Western strains, which helps explain Turkey's low rates of GC relative to countries such as Japan and Korea (Saribasak et al. 2004). In agreement with *H. pylori*'s high rate of rearrangement in the *Cag*PAI, *CagA*-positive and *CagA*-negative strains with the same genetic fingerprint are often cultured from the same individual (Morales-Espinosa et al. 1999). This suggests that during prolonged infection, *CagA*-positive strains can excise the *Cag*PAI to create isogenic *CagA*-negative strains that may remain a minority, disappear, or become the majority. Because *CagA* status is used frequently as a genetic marker, this within-host variation could contribute to the geographic variation in *CagA*-positive/negative strains; diet, the microbiome, and host genetic factors could play an important role in determining frequency of *CagA* conversion.

## Conclusions/future directions

Clinical disease caused by *H. pylori* is predicted to stem from biochemical changes in stomach physiology, such as changes in pH, nutrient supply, and physical alteration; however, symptoms for disease are only present in 20% of infected hosts (Blaser 1999). Disease therefore appears to be an exception to the rule, and severity appears to be directly related to the combination of duration of infection and intensity of the host's inflammatory response (correlated with bacterial density). A multitude of factors, such as CagA status, dietary habits, and host susceptibility, are known to trigger the inflammatory response, but as a generalized statement heightened inflammation increases risk of disease. With current research focusing on factors that exacerbate the inflammatory response, researchers are now working toward a better understanding of the transition of *H. pylori* along the parasite–mutualist continuum (Fig.1). This understanding will increase success in eradication for high-risk populations, while potentially maintaining the bacteria in low-risk populations, or specifically targeting more pathogenic strains. Given the recent discussion surrounding the active eradication of *H. pylori* from the human population (Lee et al. 2013), it is more important than ever to critically evaluate its role within the microbiome. The long history of coevolution between this bacteria and its human host (Kodaman et al. 2014) suggests it is particularly pertinent to address why these bacteria have had such a successful history and whether any benefits conferred in the past have now been ameliorated (Blaser 1999).

From a public health perspective, eradication is effective and realistic for the resources available; this is likely the best strategy for high-risk populations where gastric disease is common. The shortcomings of standard *H. pylori* eradication therapy are in the antibiotics it requires. ‘Triple therapy’ of a proton pump inhibitor (PPI) in combination with two antibiotics, commonly clarithromycin and metronidazole, is standard for *H. pylori* eradication. Reported eradication rates in the early 90s were 95%, whereas a 2007 meta-analysis of over 53 000 patients showed an eradication rate of <80% (Tong et al. 2007; Dajani et al. 2013). The decreasing efficacy of antibiotic therapy makes it unrealistic to expect success with population-wide eradication. New research on quadruple therapy (one PPI and three antibiotics) had a 95.8% success rate with successful eradication in 23 of 24 patients (Thiraworawong et al. 2014). Based on trends with triple therapy, this arms race has a predictable outcome, and it is likely only a matter of time before resistance evolves to quadruple therapy. With current methods, the amount of antibiotics needed for a strictly eradication-based regimen would only promote widespread antibiotic resistance, which is already considered a threat to global security by WHO. According to the CDC's 2013 Threat Report on antimicrobial resistance in the United States, two million people are infected by antibiotic resistant organisms each year with at least 23 000 people dying from these infections (CDC Threat Report 2013). The losing battle with antibiotic resistance in traditionally antibiotic-susceptible organisms drives the need for novel strategies in approaching infectious agents such as *H. pylori*. Movement must now be made to decrease dependence on antibiotics as the sole therapeutic strategy for all microbial infections.

No currently available, alternative therapies rival the efficacy of antibiotics, but current research on managing microbiomes and the protective effects from pre- and probiotic consumption continues to offer promise (Tong et al. 2007). Probiotics, defined as microorganisms with potentially beneficial health effects, such as *Lactobacilli,* secrete antimicrobial compounds that have been shown to inhibit or kill *H. pylori in vitro*. For example, supplementing standard triple therapy with *Lactobacilli* increased treatment efficacy from 71 to 81%, and *Bifidobacterium infantis* supplements to the standard therapy increased eradication from 68.9 to 83% while reducing antibiotic-related side effects (Lesbros-Pantoflickova et al. 2007; Tong et al. 2007; Dajani et al. 2013). One review found 7 of 9 human studies showed a decrease in *H. pylori* density and improvement in *H. pylori* gastritis with probiotic administration (Lesbros-Pantoflickova et al. 2007). Another study found *Lactobacillus plantarum* secretes factors that modulate inflammation during *H. pylori* infection (Thiraworawong et al. 2014). The preliminary research on probiotic treatments has provided inconsistent results with varying degrees of efficacy, and no study has been able to demonstrate *H. pylori* eradication *in vivo* from probiotics alone (Lesbros-Pantoflickova et al. 2007; Dajani et al. 2013). However, the studies demonstrating reduction of colony density and decreases in gastric inflammation (Pacifico et al. 2014) are indicative of the potential for alternative treatments through gut microbiome management as opposed to strict eradication. It is also unknown how these results reflect ‘normal’ levels of dietary probiotic intake, and more research needs to be conducted on long-term dietary supplementation of probiotics through fermented foods. Work on mouse models is beginning to uncover complex interactions between *H. pylori* and other bacterial species commonly found in the human gut, such as *Clostridium difficile* (Rolig et al. 2013), suggesting an intriguing link between dysbiosis and *H. pylori* -mediated disease.

The research presented by Kodaman et al. (2014) on human and *H. pylori* coevolution introduces the possibility of managing *H. pylori* -induced gastric disease by ‘matching’ a host and bacterial strain based on common ancestral association (i.e., artificially recreating the low-risk, genetic association between host and strain such as that observed in the case of the ‘African Enigma’). If more compatible strains are also able to outcompete less compatible strains within the host, such treatment could offer sustained health benefits. However, to do this successfully, further information about the interaction and the particular regions of the host and bacterial genome for which ancestry is most important would need to be obtained. Furthermore, to better understand the mutualistic role of *H. pylori* in low-risk, *H. pylori* -positive cohorts, more research needs to be performed to quantify the health benefits (Table1). It is possible that *H. pylori* constitutes a valuable, albeit mismanaged, member of the healthy gut microbiome and requires a specific host environment to confer benefit to the host. Any considerations of implementing a treatment regimen that uses *H. pylori* as a probiotic immediately encounters several major obstacles: [I] individualizing treatments for each patient would likely require extensive on-site technology (this complication is particularly difficult as the observed benefits of *H. pylori* colonization are most valuable in low-socioeconomic areas where medical resources are already scarce); [II] The high rates of recombination and natural competence observed in *H. pylori* would defy any attempt at maintaining a defined host–pathogen association, especially in ethnically diverse areas; [III] The mechanism for the development of carcinogenic activity by *H. pylori* is still largely uncontrollable and unpredictable. Until this mechanism is better understood, it would be unwise and unethical to propagate a known carcinogen. However, the use of recombinant genomics increases the possibility of ‘designer’ probiotics made from incorporating protective gene sequences from *H. pylori* into avirulent species to remove the risk involved with *H. pylori* infection.

Other alternatives for *H. pylori* therapy include vaccines. As a low-cost and easily distributed prophylaxis, vaccines would be ideal and have even had success in animal models through memory T-cell activation. However, high mutation rate and other immunomodulatory mechanisms used by *H. pylori* to persist in the gastric niche make it more resistant to immune response (Algood and Cover 2006; Ayala et al. 2014). Intragastric ultraviolet light phototherapy has also been effective with a >97% reduction in bacterial load of the antrum, although urea test results indicated bacteria tend to repopulate within a few days after illumination (Ayala et al. 2014). Bacteriophage therapy also holds potential to be a viable alternative to antibiotic treatment due to the specificity of many phages on their hosts compared to the wide-ranging effects of antibiotics. A recent study characterizing an *H. pylori -*specific bacteriophage KHP30 revealed a broad host range (KHP30 was able to infect 28 of 44 different *H. pylori* strains), stability over a wide range of pH from 2.5 to 10 (with the pH of the solution unchanged suggesting stability of the phage), and pseudolysogenic activity based on episomal and plasmid DNA that does not incorporate into the genome (Uchiyama et al. 2013). As the first *H. pylori* -specific phage to be characterized, the broad host range and pH stability are hopeful attributes for bacteriophage therapy, and further research needs to be performed to understand the capacity for bacterial modification/elimination *in vivo*. Finally, new developments in fecal transplants have also been shown to restore intestinal microbiotal balance in patients with *Clostridium dificile* infection, with ≈ 90% of patients showing recovery (Bakken et al. 2011). This method has not yet been extended to *H. pylori* infection, but based on evidence of competitive inhibition through probiotics, there is potential that the same principles will apply. These prospects provide exciting new directions for preventative health care but would require careful monitoring and should be approached with caution due to the extensively documented carcinogenic properties of *H. pylori*.

## Data archiving statement

There are no novel data associated with this review.
